# Trypsin promotes porcine deltacoronavirus mediating cell-to-cell fusion in a cell type-dependent manner

**DOI:** 10.1080/22221751.2020.1730245

**Published:** 2020-02-24

**Authors:** Yue-Lin Yang, Fandan Meng, Pan Qin, Georg Herrler, Yao-Wei Huang, Yan-Dong Tang

**Affiliations:** a State Key Laboratory of Veterinary Biotechnology, Harbin Veterinary Research Institute of Chinese Academy of Agricultural Sciences, Harbin, People’s Republic of China; b Institute of Preventive Veterinary Medicine and Key Laboratory of Animal Virology of Ministry of Agriculture, College of Animal Sciences, Zhejiang University, Hangzhou, People’s Republic of China; c Institute for Virology, University of Veterinary Medicine Hannover, Foundation, Hannover, Germany

**Keywords:** PDCoV, trypsin, entry, virus release, cell-to-cell fusion

## Abstract

Porcine deltacoronavirus (PDCoV) is a newly emerging threat to the global porcine industry. PDCoV has been successfully isolated using various medium additives including trypsin, and although we know it is important for viral replication, the mechanism has not been fully elucidated. Here, we systematically investigated the role of trypsin in PDCoV replication including cell entry, cell-to-cell membrane fusion and virus release. Using pseudovirus entry assays, we demonstrated that PDCoV entry is not trypsin dependent. Furthermore, unlike porcine epidemic diarrhea virus (PEDV), in which trypsin is important for the release of virus from infected cells, PDCoV release was not affected by trypsin. We also demonstrated that trypsin promotes PDCoV replication by enhancing cell-to-cell membrane fusion. Most importantly, our study illustrates two distinct spreading patterns from infected cells to uninfected cells during PDCoV transmission, and the role of trypsin in PDCoV replication in cells with different virus spreading types. Overall, these results clarify that trypsin promotes PDCoV replication by mediating cell-to-cell fusion transmission but is not crucial for viral entry. This knowledge can potentially contribute to improvement of virus production efficiency in culture, not only for vaccine preparation but also to develop antiviral treatments.

## Introduction

Porcine deltacoronavirus (PDCoV) (genus *Deltacoronavirus*; family *Coronaviridae*) is a newly emerging swine pathogen [1–4]. Acute cases of PDCoV infection exhibit watery diarrhea in sows and nursing piglets, resulting in severe gastrointestinal disease which may have a lethal outcome [4–6]. PDCoV poses a major threat to the swine industry, and is currently epidemic in several countries; first reported in Hong Kong in 2012 [7], it has since been found in the United States [8–10], Canada [11], South Korea [12–15], mainland China [16–19], Thailand [20–22] and Vietnam [23]. Importantly, porcine aminopeptidase N (pAPN) has been reported to serve as a functional receptor for PDCoV in two recent studies [24,25], and the virus may engage APN from diverse species to facilitate its interspecies transmission [25]. Recently, PDCoV has been reported to successfully infect chickens and calves [26,27]. Thus, PDCoV must be studied more extensively to better understand its emergence, lifecycle, evolution and pathogenesis in order to facilitate future control of the virus.

Despite the many reports of PDCoV outbreaks, very few viruses have been successfully recovered, showing the difficulty of virus isolation [4,28]. PDCoV was first isolated in swine testicular (ST) and LLC porcine kidney (LLC-PK) cells by adding trypsin or pancreatin [28]. Although trypsin was used for PDCoV isolation and propagation, its role in the virus lifecycle remains unclear. To address this aspect, we evaluated the importance of trypsin for the PDCoV infection in two different cell lines (LLC-PK and ST).

We developed a PDCoV pseudotype virus system to investigate the impact of trypsin on viral entry. Our findings indicate that PDCoV entry was not promoted by trypsin. We further illustrate that virus release was also not influenced by this protease. Our findings provide evidence that trypsin plays an important role in PDCoV-mediated cell-to-cell membrane fusion, which facilitates virus spread.

## Materials and methods

### Cells, virus, reagent and plasmids

The ST cell line (swine testicle; ATCC CRL1746), LLC-PK cell line (porcine kidney; ATCC CL-101), HEK293T and HEK293 (human embryonic kidney) cells were maintained in DMEM (Gibco, USA) with 10% foetal bovine serum (HyClone, USA). The trypsin used in this study was purchased from Gibco (LOT: 1968166). The HEK293-APN cell line (stably expressing pAPN) was generated by the piggyBac (PB) transposon system [29]. pAPN was amplified by PCR including a FLAG tag in the forward primer (F: CATAGAAGATTCTAGACACCATGGATTACAAGGACGACGATGACAAGgccaagggattctacatttc, R: ATTTAAATTCGAATTCttagctgtgctctatgaacca) and then cloned into the pB513B vector to generate pB513B-APN (System Biosciences, Mountain View, USA) [29]. Then, HEK293 cells were co-transfected with 3 μg pB513B-APN and 1 μg helper vector expressing PB transposase (System Biosciences, Mountain View, USA). Forty eight hours later, cell media were replaced with growth media containing 1 µg/ml puromycin, (Gibico, USA) and replaced every 2 days. The PDCoV S gene was cloned into pCAGGS-HA by the following primers with EcoR I and Xho I (F: CTGAATTCCTCGAGATGCAGAGAGCTC, R: AACTCGAGCTACCATTCCTTAAACTTAAAGG). PDCoV Chinese “Hunan” strain was used as in our previous described study [30]. PDCoV was isolated and prepared in LLC-PK cells (less than 15 passages) in the presence of 5 μg/ml trypsin and without foetal bovine serum. PDCoV in the current study was passaged fewer than 10 times, and titred by plaque assay in ST cells. Briefly, when ST cells reached up to 100% confluence, they were washed with PBS three times and subsequently infected with PDCoV in the presence of 5 μg/ml trypsin. Two hours later, the cells were overlaid with 2% low-melting agarose and maintained with 5 μg/ml trypsin in DMEM at 37°C with 5% CO_2_ for 3–4 days. The cells were then stained with 0.5% crystal violet, and the plaques were counted.

### Pseudovirus entry assay

The PDCoV pseudovirus was produced in HEK293T cells as previously described [31]. Briefly, HEK293T cells were seeded in 6-well plates and when cell confluency reached 30–40%, HIV-1 based luciferase reporter plasmids were co-transfected (by calcium phosphate) with the helper plasmids psPAX2 (Addgene, USA) and PDCoV-S to generate pseudotyped viruses. After 8 h, cells were washed with PBS and then serum-free medium was added. The pseudovirus in the supernatant was collected at 48 h post-transfection, and 100 μl was used to infect LLC-PK and ST cells. These were washed and subjected to luciferase analysis at 24 h post-infection (hpi).

### PDCoV entry assay

LLC-PK and ST cells were seeded in 6-well plates, and when they reached 90% confluence, cells were infected at MOI = 0.1 of PDCoV in the presence of indicated trypsin concentrations (5, 10, 20 and 200 μg/ml) at 37°C with 5% CO_2_. Two hours later, the cells were washed three times with PBS, and RNA was extracted and quantified by qPCR as described previously [24].

### Releasing assay

*Assay 1*. LLC-PK and ST cells were infected with PDCoV at a multiplicity of infection (MOI) of 10 in the presence or not of trypsin, and the virus released to the supernatant was collected at 12 and 24 hpi. Samples were centrifuged at 12,000× *g* for 10 min at 4°C to remove cell debris, and centrifuged again at 20,000× *g* for 2 h at 4°C to pellet the virions. Meanwhile, the virus-infected cells were washed once with PBS and then lysed in radio immunoprecipitation assay (RIPA) lysis buffer containing a protease inhibitor cocktail (Roche, USA). Floating and necrotic cells were centrifuged at 5000× *g* for 10 min at 4°C, and pelleted cells were included in the experiment. N protein-specific antibody was prepared and stored in our lab. The virions in both the supernatant and cell lysate were analyzed by western blot.

*Assay 2*. LLC-PK cells were infected with PDCoV (MOI = 0.1 and 1) in 5 μg/ml trypsin for 24 h, and the cells were further cultivated without trypsin for 48 h, then infected cells were treated with indicated concentration (5 and 20 μg/ml) of trypsin at 37°C for 5 min. Floating and necrotic cells were centrifuged at 5000× *g* for 10 min at 4°C, and pelleted cells were included in the experiment. Virus titre was quantified by plaque assay as described above.

### Immunofluorescence assay

LLC-PK and HEK293-APN cells were plated in 24-well plates, and when confluency reached 90%, cells were washed three times with PBS and infected with PDCoV at different MOI in the presence or not of trypsin. After 12 h, cells were fixed in 4% paraformaldehyde for 1 h, washed three times with PBS and then permeabilized with 0.2% triton X-100 for 1 h. After washing with PBS three times, cells were blocked with 1% BSA for 2 h, then incubated for 1 h at room temperature with a monoclonal antibody specific for the PDCoV N protein. Alexa Fluor 568-conjugated goat anti-mouse IgG (Sigma, USA) was used as the secondary antibody; for nuclear visualization, cells were stained with DAPI (Sigma, USA).

### Cell-to-cell membrane fusion assay

HEK293-APN cells were first plated in 6-well plates, and when confluency reached 90%, cells were transfected with the indicated plasmids: HEK293-APN effector cells were co-transfected with 1 μg pGL5-Luc (Promega, USA) and 16 μg PDCoV-S; target cells were transfected with 6 μg PBind-Id (Promega, USA) and 6 μg PACT-Myod (Promega, USA). PBind-Id and PACT-Myod generate fusion proteins containing the DNA-binding domain of GAL4 and the activation domain of VP16, respectively. The pGL5-Luc vector contains five GAL4 binding sites upstream of a minimal TATA box, which in turn, is upstream of the firefly luciferase gene. PBind-Id and PACT-Myod collaborate to initiate firefly luciferase expression of the pGL5-Luc vector only if cell fusion occurs. After 18 h, both effector and target cells were detached with trypsin and washed with PBS for three times then the pellet was resuspended with culture medium and mixed at a 1:1 ratio, and seeded into fresh 96-well plates. After attachment, medium was replaced with or without trypsin, and luciferase activities were measured after two days of co-cultivation.

### PDCoV susceptibility assay

After seeding in 6-well plates and the confluency of each cells reached around 90%, PDCoV was used to infect LLC-PK (MOI = 0.5, 1 and 10) and ST cells (MOI = 1, 2 and 5), washed twice with PBS at 2 hpi, and then medium supplemented or not with 5 μg/ml trypsin was added. Infected cells were lysed and subjected to western blot at 8, 12 and 24 hpi.

### PDCoV S protein cleavage assay

Cleavage assay of S protein in virions: PDCoV virions were purified by centrifugation at 20,000× *g* for 2 h at 4°C, and virions were incubated with the indicated concentrations (1, 5, 10, 20 μg/ml) of trypsin at 37°C for 2 h. N protein was used as a virus loading control.

Cleavage assay of S protein in virus infected cells: LLC-PK and ST cells were infected with PDCoV (MOI = 0.1 and 10, respectively) in 5 μg/ml trypsin, and incubated for 24 h in order to increase virus replication and bring S protein to a detectable level. Then, the cells were further cultivated without trypsin for 24 h, and both cell types were treated with the indicated concentrations (5, 50, 100, 200 μg/ml) of trypsin at 37°C for 2 h. Floating and necrotic cells were centrifuged at 5000× *g* for 10 min at 4°C, and pelleted cells were included in the experiment. N protein was used as a virus loading control.

### Establishment of cell-to-cell transmission assay

LLC-PK cells of 2.5 × 10^6^ were seeded in a 10-mm petri dish, and when the cells reached confluence, they were inoculated with PDCoV at MOI = 1 in 5 μg/ml of trypsin and incubated at 37°C in 5% CO_2_. These virus-infected cells were defined as *effector cells*. Other LLC-PK cells were seeded in 24-well plates at a density of 1.0 × 10^5^ cells/well for 24 h, and then labelled with cell tracker dye deep red (Invitrogen), which can label the cytoplasm of living cells. These naïve, pre-labelled cells were defined as *target cells*. At 24 hpi, the effector cells were detached and washed with fresh culture medium twice to remove residual trypsin. Afterwards, the collected effector cells were added directly to the target cells already growing in 24-well plates (contact cell model). Simultaneously, the same number of effector cells as mentioned above were seeded on trans-well filters (Corning, 6.5 mm, 0.4 μm pore size) at a density of 0.3 × 10^5^ cells. The filters were suspended in wells in a 24-well plate already containing target cells (uncontact cell model). In both infection models, medium supplemented or not with 5 μg/ml trypsin was added. After 48 h of interaction between effector and target cells, infection in target cells was detected as the presence of viral N protein by immunofluorescence assay, and both target and effector cells were collected for viral titration.

### Statistical analysis

Origin GraphPad Prism 8.0 software was used for all graphical representations. Statistical significance was analyzed by one-way-ANOVA and Tukey’s multiple comparison test or the independent Student’s *t*-test. All *p* values < 0.05 were considered statistically significant.

## Results

### Trypsin significantly promotes PDCoV replication in LLC-PK cells but not ST cells

**Figure 1.**
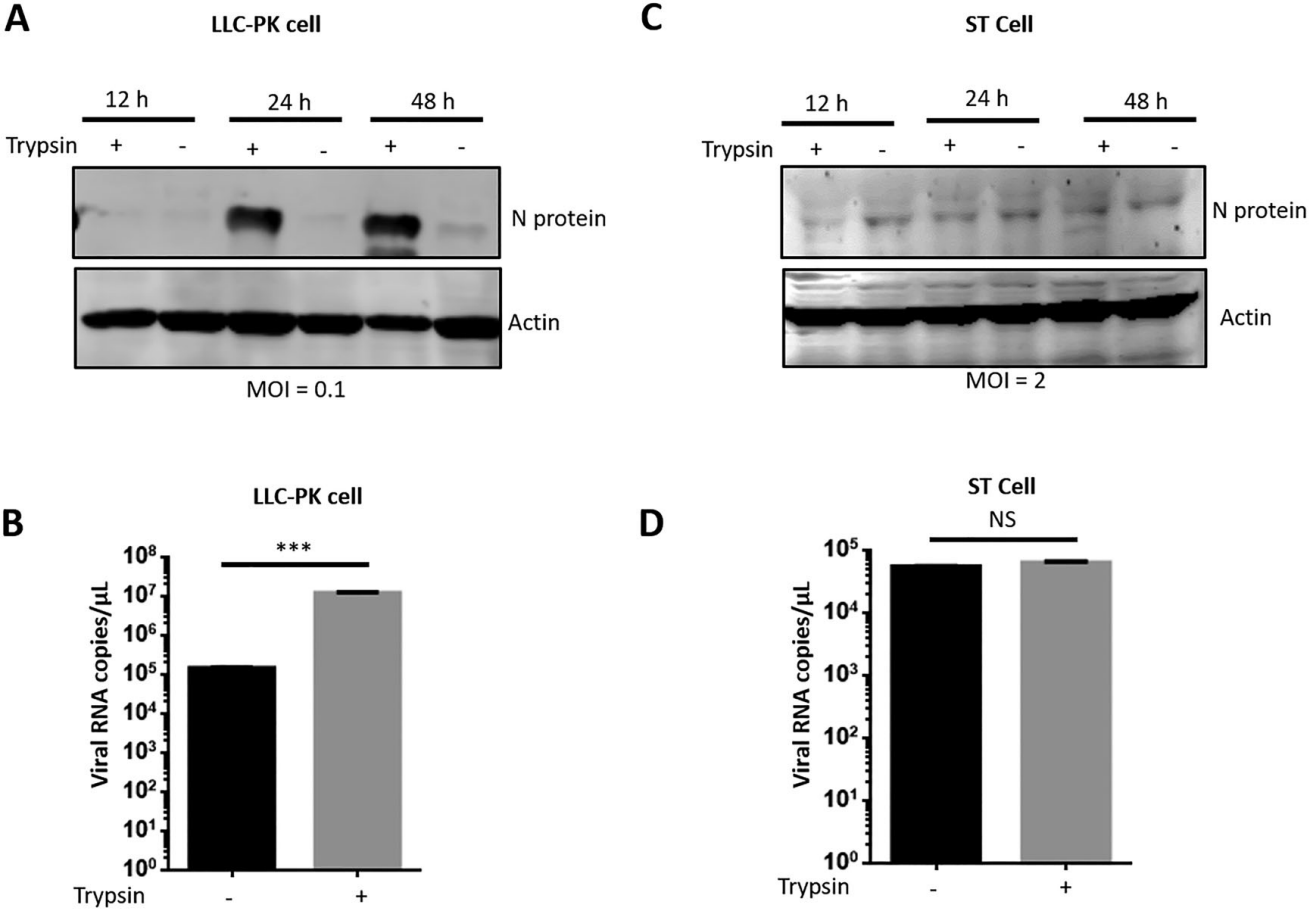
Trypsin significantly promotes PDCoV replication in LLC-PK cells but not ST cells. (A) LLC-PK cells were infected with PDCoV at an MOI of 0.1 in the presence or absence of 5 μg/ml trypsin, and then cells were collected at indicated time points. After cell lysis, PDCoV N proteins were analyzed by western blot. (B) Viral RNA was collected at 48 hpi from the experiment in (A) and quantified by qPCR. (C) ST cells were infected with PDCoV at an MOI of 2 in the presence or absence of 5 μg/ml trypsin, and then cells were collected at indicated time points. After cell lysis, PDCoV N proteins were analyzed by western blot. (D) Viral RNA was collected at 48 hpi from the experiment in (C) and quantified by qPCR. Each experiment was repeated at least three times. Error bars represent the standard error of the mean (SEM). *** stand for *p* < 0.001, NS: no significant difference.

In previous studies, PDCoV was successfully isolated in ST or LLC-PK cells by adding trypsin to the medium [4,16,28]. However, the mechanism for how trypsin promotes PDCoV replication is unknown. To explore whether it is essential for PDCoV replication, we first infected LLC-PK cells at a low virus/cell ratio (MOI = 0.1) and determined the virus yield in the presence or absence of trypsin by western blot at different times post-infection. Only a faint band of viral N protein was detectable at 12 hpi. PDCoV N protein production was significantly enhanced at 24 or 48 hpi in trypsin-treated samples as compared to the untreated control (Figure 1(A)). In the absence of the exogenous protease, only a weak band of N protein was detected at 48 hpi, consistent with previous reports [4,28]. We further quantified viral titre by qPCR as described previously [24], revealing that trypsin significantly promoted PDCoV replication in LLC-PK cells at 48 hpi (Figure 1(B)).

Next, we evaluated whether trypsin was essential for PDCoV replication in ST cells. It was difficult to detect N protein at a low MOI (data not shown), and increasing the infectious dose up to MOI = 2 resulted in detection of only a weak band (Figure 1(C)). However, to our surprise, PDCoV replication was no different in ST cells regardless of the presence or absence of trypsin (Figure 1(C, D)). Taken together, these results indicate that trypsin significantly promotes PDCoV replication in LLC-PK cells but not in ST cells.

### Trypsin does not affect PDCoV entry of LLC-PK cells and ST cells

**Figure 2.**
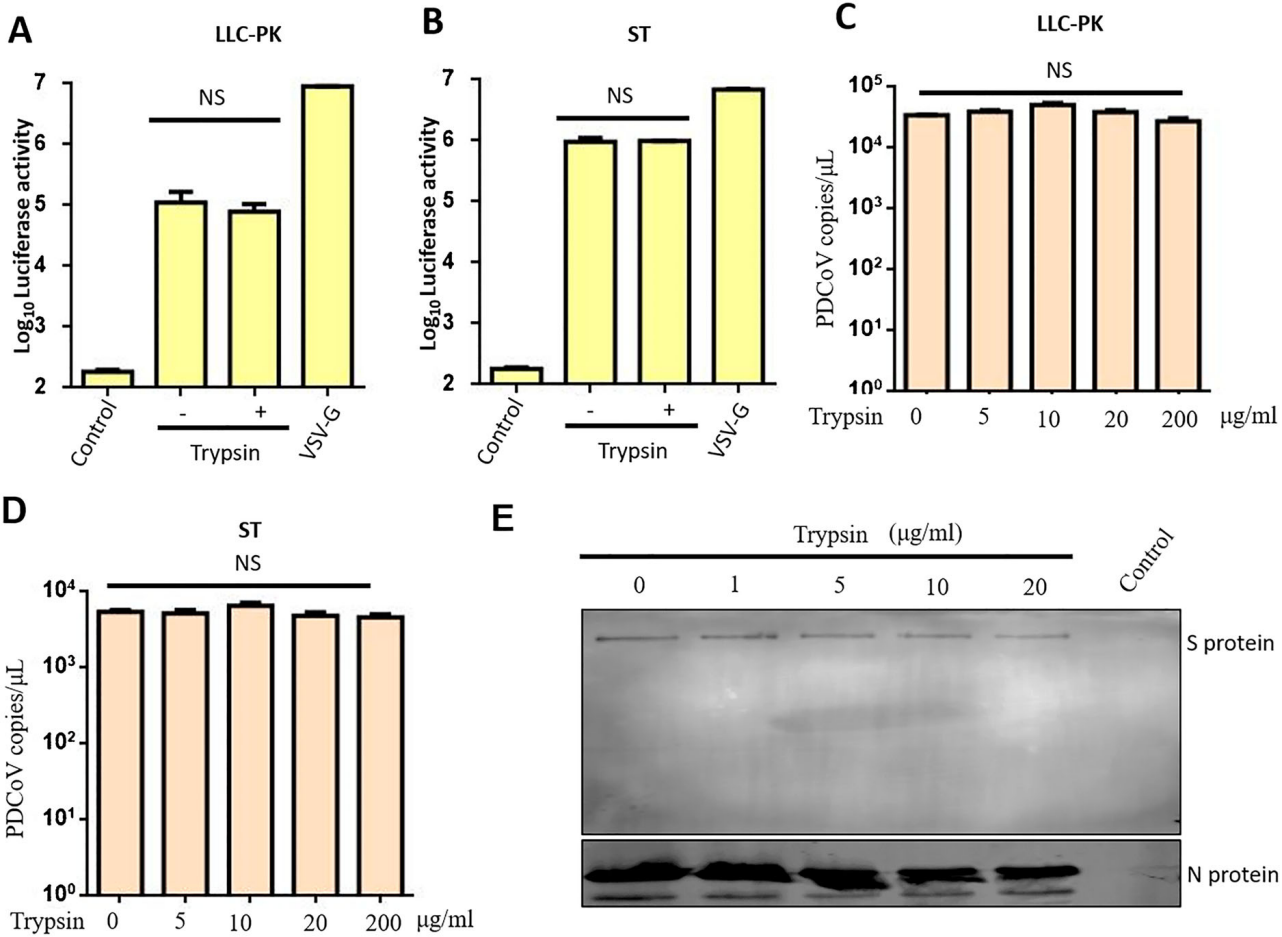
Trypsin doesn’t affect PDCoV entry by pseudovirus or real virus. Entry into (A) LLC-PK and (B) ST cells was tested using pseudotyped retroviruses displaying the PDCoV spike. Recombinant viruses containing luciferase were generated in HEK293T cells and then used to infect different cell lines in the presence or absence of 5 μg/ml trypsin. VSV-G pseudovirus was used as a positive control, and non-enveloped packaging group was a negative control. Twenty four hours later, cells were washed and lysed for luciferase activity detection. Entry of real PDCoV into (C) LLC-PK and (D) ST cells was quantified by qPCR. LLC-PK and ST cells were infected with MOI = 0.1 of PDCoV in the presence of the indicated trypsin concentration, and 2 h later, cells were washed and RNA was extracted and quantified by qPCR. (E) Cleavage status of S protein by indicated concentration of trypsin (1, 5, 10, 20 μg/ml). PDCoV virions were purified by centrifugation at 20,000× *g* for 2 h at 4°C, and virions were incubated with the indicated concentration of trypsin at 37°C for 2 h. N protein was used as a virus loading control. The above experiments were repeated at least three times. Error bars represent the SEM. NS: no significant difference.

To elucidate which step of the viral replication cycle is being affected by trypsin in LLC-PK or ST cells, we first considered the initial stage of infection. To assay viral entry, we used a pseudovirus approach in LLC-PK. Briefly, 100 μl of lentivirus-based pseudovirus containing the S protein of PDCoV was incubated with each of the two cell types for 24 h in the presence or absence of trypsin, washed three times with PBS and subjected to luciferase analysis. VSV-G pseudovirus was used as a positive control, and non-enveloped packaging group as a negative control. There was no significant difference in luciferase activity in the presence or absence of trypsin treatment in LLC-PK cells (Figure 2(A)). We got a similar result when ST cells were infected with pseudovirus (Figure 2(B)). This result indicates that trypsin treatment does not promote PDCoV-S protein-mediated entry of pseudoviruses into LLC-PK and ST cells. Next, we wanted to know whether trypsin treatment influences entry of real virus, and whether PDCoV entry was influence by various concentrations of trypsin. LLC-PK cells and ST cells first were infected at MOI = 0.1 with PDCoV in the presence of the indicated concentration of trypsin, and at 2 hpi, entry of PDCoV was quantified by qPCR. The results indicated that entry of real PDCoV into both cell lines was not influenced by trypsin, despite increasing the concentration of trypsin up to 200 μg/ml in both cells (Figure 2(C, D)). Cleavage of the Coronavirus S protein by trypsin always plays a determinant role in virus entry. To test whether S protein was cleaved by trypsin in the current study, we first purified PDCoV virions and then treated them with different concentrations (1, 5, 10, 20 μg/ml) of trypsin at 37°C for 2 h. We did not obviously detect S protein cleavage (Figure 2(E)); thus, we think trypsin is not involved in the PDCoV entry process in LLC-PK or ST cells.

### Trypsin does not affect PDCoV egress from infected LLC-PK cells or ST cells

**Figure 3.**
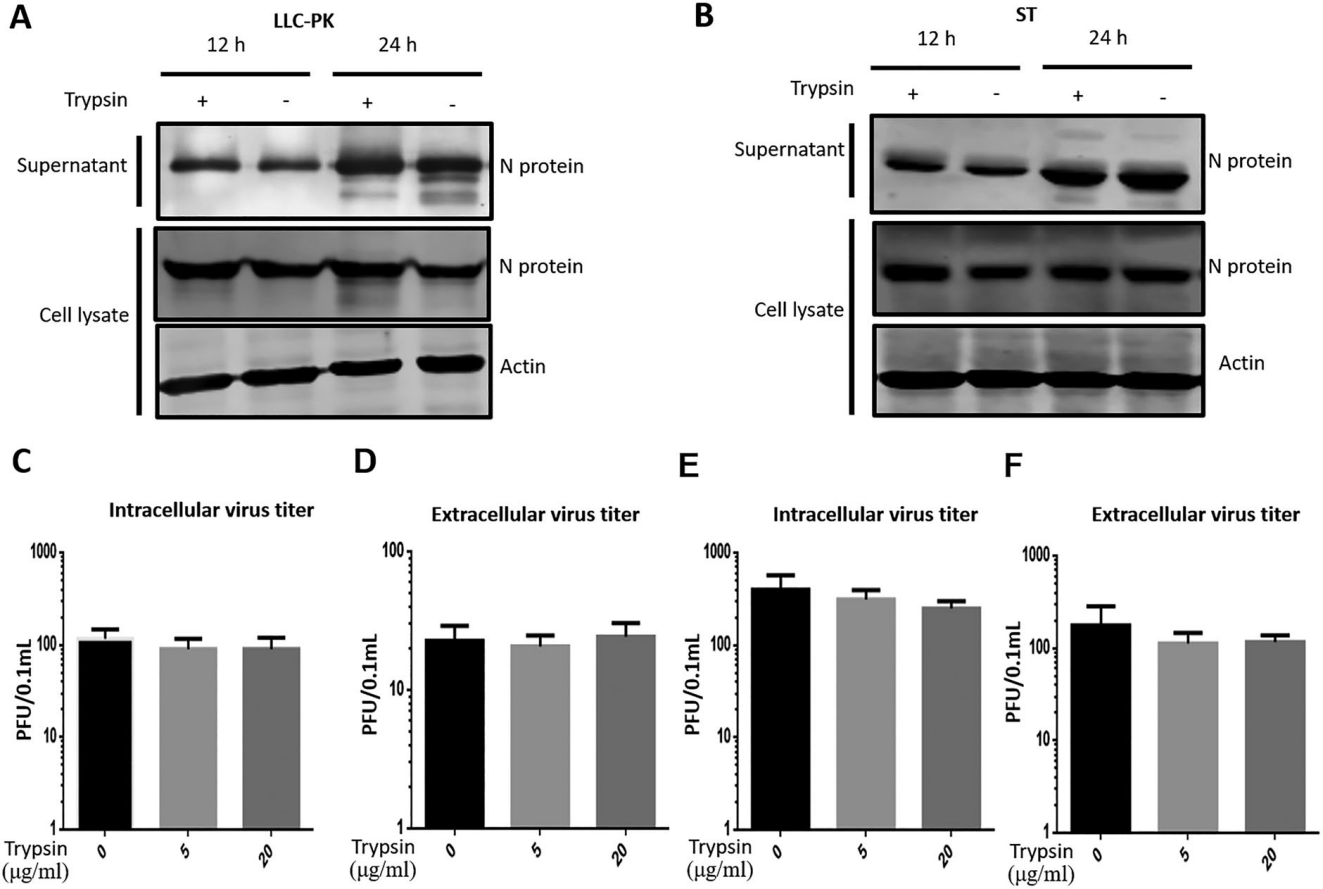
Trypsin doesn’t affect PDCoV release. Release of PDCoV from (A) LLC-PK and (B) ST cells was analyzed with an MOI of 10 in the presence or absence of trypsin (5 μg/ml). The supernatant and the cell pellets were collected at 12 and 24 hpi respectively, and expression of viral N protein in both the supernatant and cell lysate was analyzed by western blot. LLC-PK cells were infected with PDCoV at MOI = 0.1 (C–D) or MOI = 1 (E–F) and treated with trypsin (5 μg/ml) for 24 h to increase virus replication, and the cells were further cultivated without trypsin for 48 h, then both cells were treated by indicated concentrations (5 and 20 μg/ml) of trypsin at 37°C for 5 min. Virus was titrated in the cells (C and E) and supernatant (D and F) using plaque assay. Experiments were repeated at least three times. Error bars represent the standard error of the mean. NS = no significant difference.

Next, we analyzed whether trypsin supports the egress of PDCoV from infected cells, as has been shown previously for PEDV infection [32]. To this end, we infected LLC-PK and ST cells at a high multiplicity (MOI = 10) to limit cell-to-cell spread of infection. We also demonstrated that when LLC-PK and ST cells were infected at a low multiplicity, the PDCoV virus was more prone to cell-to-cell transmission rather than releasing the viruses (Figure S1). In order to differentiate between intracellular virus and virus released from infected cells, the cell lysates and supernatants were collected separately. The amount of virus in cell lysates or in the supernatant fraction at 12 and 24 hpi was not significantly altered by the presence of trypsin (5 μg/ml) in either cell type (Figure 3(A, B)). To further confirm this, we performed a release assay in LLC-PK cells as described previously [32]. LLC-PK cells were infected with PDCoV (MOI = 0.1 and 1) with trypsin for 24 h (to increase virus replication), the cells were further cultivated without trypsin for 48 h, then both cells were treated with the indicated concentrations (5 and 20 μg/ml) of trypsin at 37°C for 5 min. There was no significant difference in intracellular virus titre (Figure 3(C, E)) or titre in the supernatant (Figure 3(D, F)), regardless of trypsin treatment. These results indicate that unlike PEDV, the release of PDCoV is not substantially enhanced by the addition of trypsin [32].

### Trypsin enhances PDCoV cell-to-cell spread in LLC-PK cells by promoting membrane fusion

**Figure 4.**
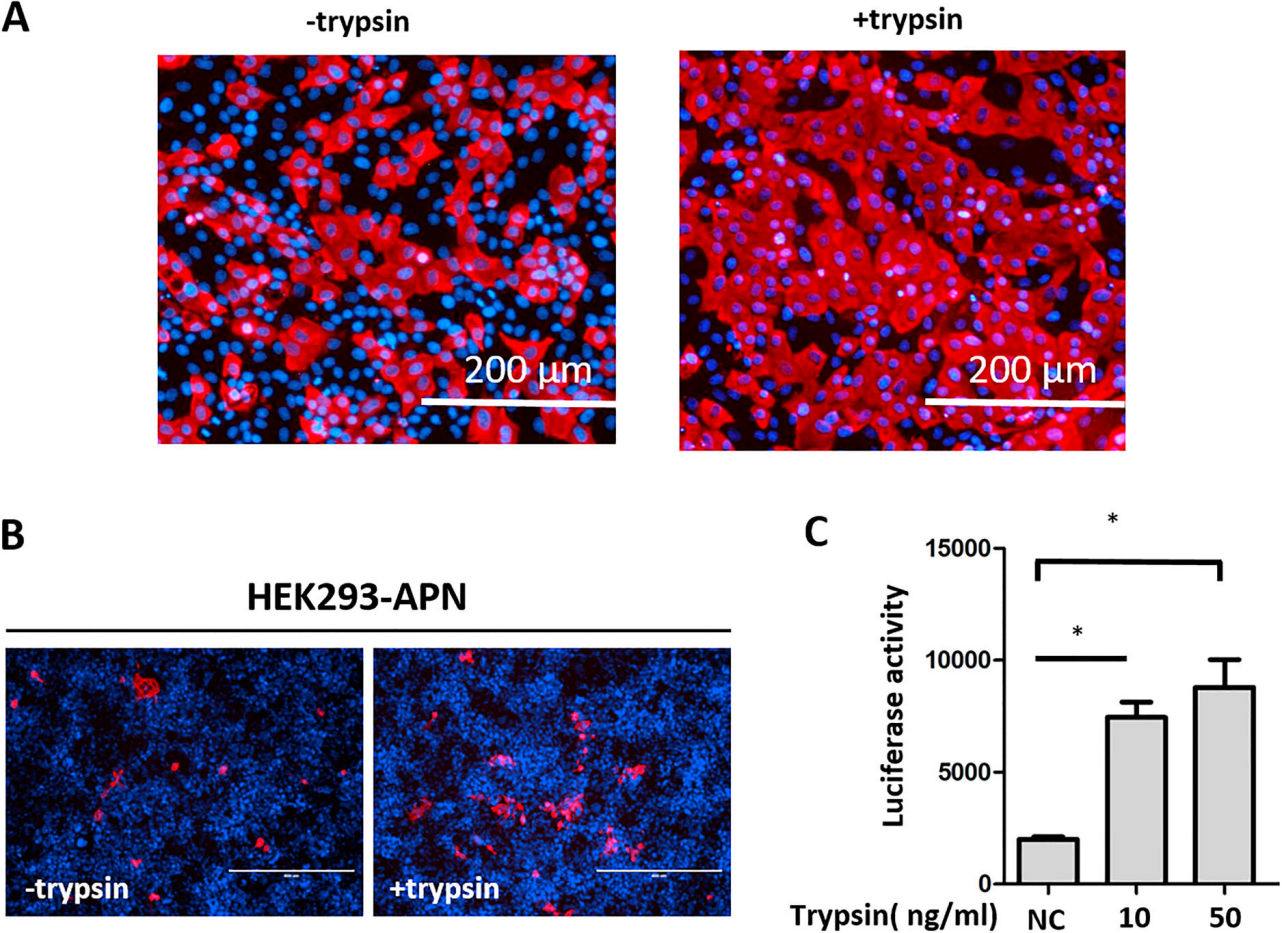
Trypsin promotes PDCoV-mediated cell-to-cell membrane fusion. (A) LLC-PK cells were infected with PDCoV at an MOI of 1 for 2 h, washed with PBS and cultured in the presence or absence of 5 μg/ml trypsin. An immunofluorescence assay (IFA) was performed at 12 h post-infection (hpi); PDCoV N was stained and the cell nuclei were labelled by DAPI. Scale bar = 200 μm. (B) HEK293-APN cells were infected with PDCoV at an MOI of 0.5 in the presence or absence of 0.01 μg/ml trypsin, and IFA was performed at 24 hpi. The PDCoV N protein was stained and the cell nuclei was labelled by DAPI. Scale bar = 400 μm. (C) PDCoV spike-mediated cell-to-cell membrane fusion was studied in the presence of trypsin. HEK293-APN cells were co-transfected with pBind-Id and PACT-Myod and mixed with other HEK293-APN cells co-transfected with PDCoV spike and PGL5-Luc. After attachment, cells were co-cultured in fresh media containing 10 or 50 ng/ml trypsin, or no trypsin (NC). After 48 h, cell-to-cell membrane fusion was evaluated using luciferase activity; *: *p *< 0.05 (*t*-test). Experiments were repeated at least three times.

Next, we investigated whether trypsin promotes PDCoV replication by inducing cell-to-cell membrane fusion. We infected LLC-PK cells at an MOI of 1, and stained infected cells with antibodies directed against the N protein at 12 hpi. PDCoV induced cell fusion was detected in LLC-PK cells treated with trypsin (Figure 4(A)), indicating that the exogenous protease significantly promoted cell-to-cell membrane fusion of LLC-PK cells. To confirm this result, we used a luciferase reporter system to analyze cell-to-cell fusion with HEK293-APN cells [33–35]. In previous studies, APN has been shown to serve as a PDCoV receptor [24,25]; therefore, we stably expressed pAPN in HEK293 cells by applying the piggyBac (PB) transposon system. After having confirmed that pAPN was well expressed in HEK293 cells (data not shown), we analyzed whether PDCoV could induce cell-to-cell fusion in HEK293-APN cells at MOI = 0.5. In the presence of trypsin, several virus-infected cells were located next to each other (Figure 4(B)), their contacting cell membranes having disappeared and their nuclei gathered in large conglomerates similar to what was observed in LLC-PK cells. We next performed a cell-to-cell membrane fusion assay in HEK293-APN cells. HEK293-APN effector cells were transfected with PDCoV S plasmid and PGL5-Luc, and co-cultivated with HEK293-APN target cells transfected with pBind-Id and PACT-Myod plasmids. After mixing the effector and target cells, fresh medium with or without trypsin was added, and luciferase activity was measured after two days of co-cultivation (Figure 4(C)), revealing a dose-dependent effect. Compared to the untreated control, fusion activity was increased at 10 ng/ml, but it was most pronounced at 50 ng/ml trypsin. These results indicate that trypsin significantly increases cell-to-cell fusion activity during PDCoV infection of LLC-PK cells.

### LLC-PK cells are more susceptible to PDCoV infection than ST cells under similar condition where trypsin is supplemented in the cell culture medium

**Figure 5.**
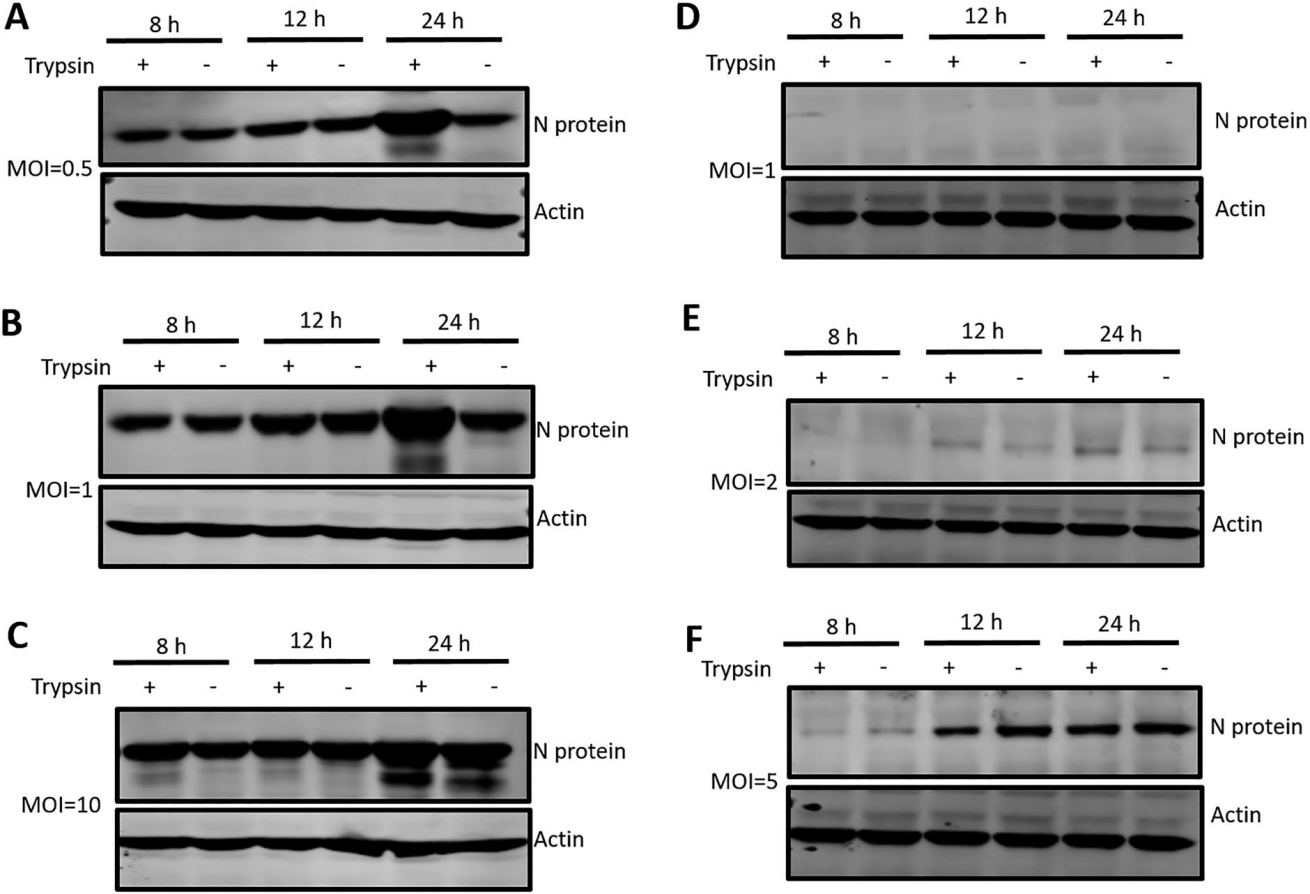
LLC-PK cells were more susceptible to PDCoV infection than ST cells. LLC-PK cells were infected with PDCoV at an MOI of (A) 0.5, (B) 1, or (C) 10, and ST cells were similarly infected at an MOI of (D) 1, (E) 2, or (F) 5. Both infected cell types were cultured in the presence or absence of 5 μg/ml trypsin and then cells were washed and lysed for western blot at 8, 12 and 24 hpi. PDCoV N proteins were analyzed with a specific antibody against N protein, and actin was used as a loading control. Experiments were repeated at least three times.

In a previous study, Hu et al. successfully isolated PDCoV in both LLC-PK cells and ST cells [28]; ST cells in general are less susceptible to PDCoV infection than LLC-PK cells. In the current study, we analyzed the susceptibility of both cell lines to PDCoV in the presence or absence of trypsin. We first infected LLC-PK cells at different MOIs from 0.5-10, and evaluated PDCoV replication by analyzing the cells at 8, 12 and 24 hpi for the presence of the viral N protein. At an MOI of 0.5 and 1, trypsin did not have an effect at 8 or 12 hpi; however, it did significantly increase virus replication at 24 hpi (Figure 5(A, B)), which indicates that trypsin promotes PDCoV replication at a late stage of the viral infection rather than at an early stage (8–12 h). At high multiplicity (MOI = 10), the enhancing effect of trypsin was less pronounced (Figure 5(C)). This demonstrated that trypsin-mediated augmentation of PDCoV infection in LLC-PK cells is strongly MOI dependent. Next, we performed the experiment in ST cells, using MOIs ranging from 0.5 to 10; when an MOI of 0.5 was applied to ST cells, no bands were detectable (data not shown). When the MOI was increased to 1 and 2, only faint viral N protein bands were observed (Figure 5(D, E)), and at an MOI = 5, we detected more robust PDCoV replication (Figure 5(F)). However, trypsin treatment did not have a noticeable effect on viral replication at the times analyzed (Figure 5(F)). Furthermore, the amount of viral N protein in ST cells at 8 hpi (MOI = 5) was much lower than that in LLC-PK cells (MOI = 0.5). The same result was obtained at MOI = 10 in ST cells (Figure 2). These results confirm that LLC-PK cells are more susceptible to PDCoV infection than ST cells, and that trypsin promotes PDCoV replication at a late stage in LLC-PK cells but not in ST cells.

### PDCoV spreading is different in LLC-PK cells and ST cells

**Figure 6.**
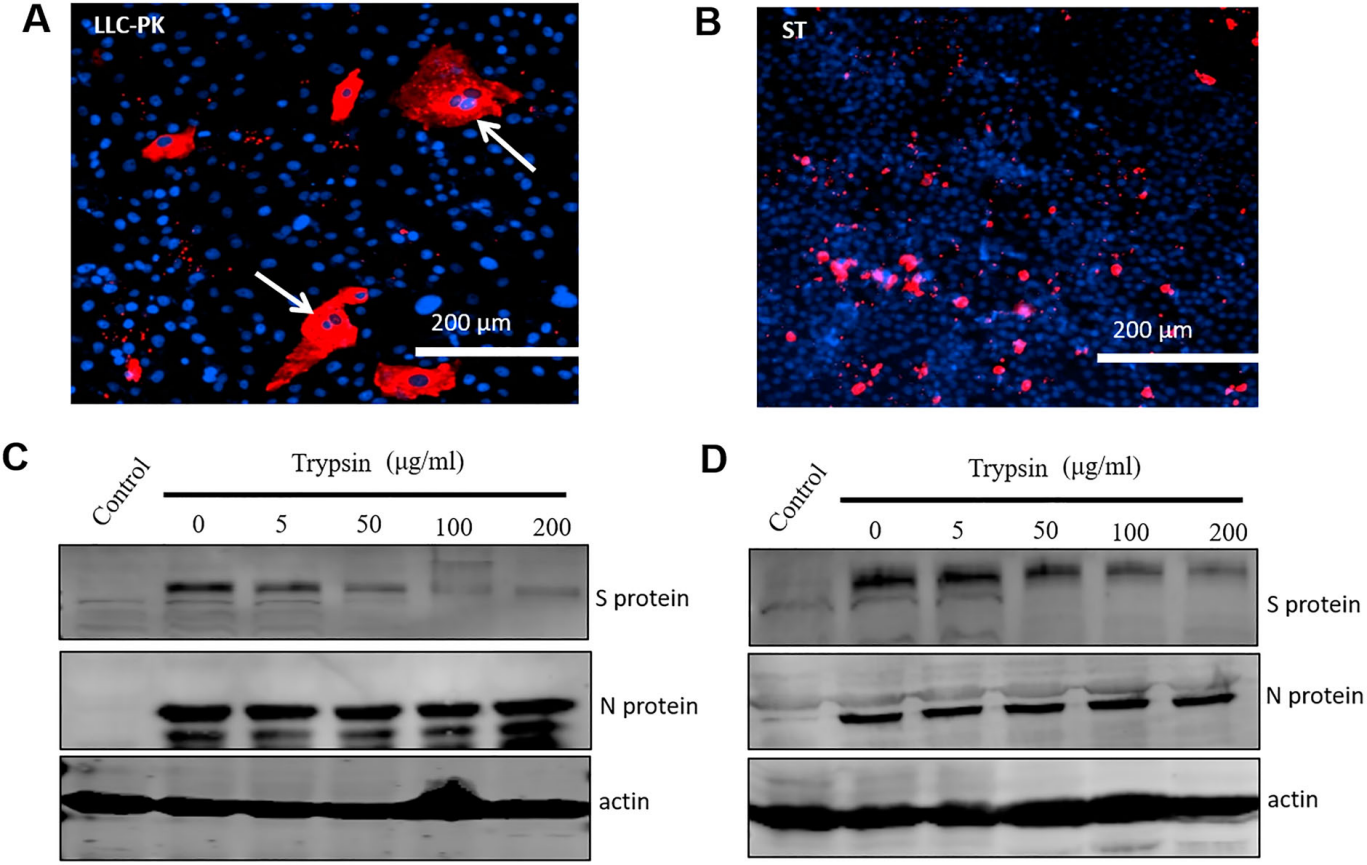
Spread of PDCoV was different in LLC-PK and ST cells. (A) LLC-PK and (B) ST cells were infected with PDCoV at a low MOI (MOI = 0.01) with trypsin (5 μg/ml), then samples were fixed and IFA performed at 48 hpi. The PDCoV N protein was stained and the cell nuclei was labelled by DAPI. Arrows indicate syncytium formation; scale bar = 200 μm. Cleavage status of S protein in (C) LLC-PK cells and (D) ST cells by indicated concentration (5, 50, 100, 200 μg/ml) of trypsin. LLC-PK cells were infected in the presence of trypsin with PDCoV (MOI = 0.1), whereas ST cells were infected with PDCoV (MOI = 10) for 24 h. In order to increase virus replication and bring S protein to a detectable level, the cells were further cultivated without trypsin for 24 h, then both cells were treated by the indicated concentration (5, 50, 100, 200 μg/ml) of trypsin at 37°C for 2 h. N protein and actin were used as a virus loading control. Experiments were repeated at least three times.

It seems that the different effects of trypsin on PDCoV replication in LLC-PK and ST cells are responsible for the different spreading patterns observed in the two cell lines. To test this hypothesis, we infected both cell types with PDCoV and performed IFA at 48 hpi to visualize cell spread. PDCoV infection in LLC-PK cells exhibited a spreading pattern consistent with cell-to-cell fusion (syncytium formation as indicated by arrows) (Figure 6(A)). However, in ST cells, PDCoV transmission was completely different, showing mainly single virus-infected cells and no obvious syncytium formation (Figure 6(B)). Taken together, the above results show that trypsin promotes PDCoV infection in LLC-PK cells by enhancing cell-to-cell fusion, whereas by contrast, trypsin does not facilitate transmission of PDCoV infection in ST cells. Coronavirus S protein cleavage by trypsin always plays a critical role for cell-to-cell fusion. To test whether cleavage of S protein by trypsin was different in LLC-PK and ST cells, we infected both cell types with PDCoV for 24 h with trypsin (to increase virus replication and bring S protein to a detectable level). The cells were further cultivated without trypsin for 24 h, then treated with indicated concentrations (5, 50, 100, 200 μg/ml) of trypsin at 37°C for 2 h. The results showed a clear cleavage of S protein in LLC-PK cells (Figure 6(C)), but was less efficient in ST cells (Figure 6(C, D)). The results indicated that differential cleavage of the S protein may be involved in the variable effects of trypsin on PDCoV replication in LLC-PK and ST cells.

### Efficiency of PDCoV spreading by cell-to-cell fusion

**Figure 7.**
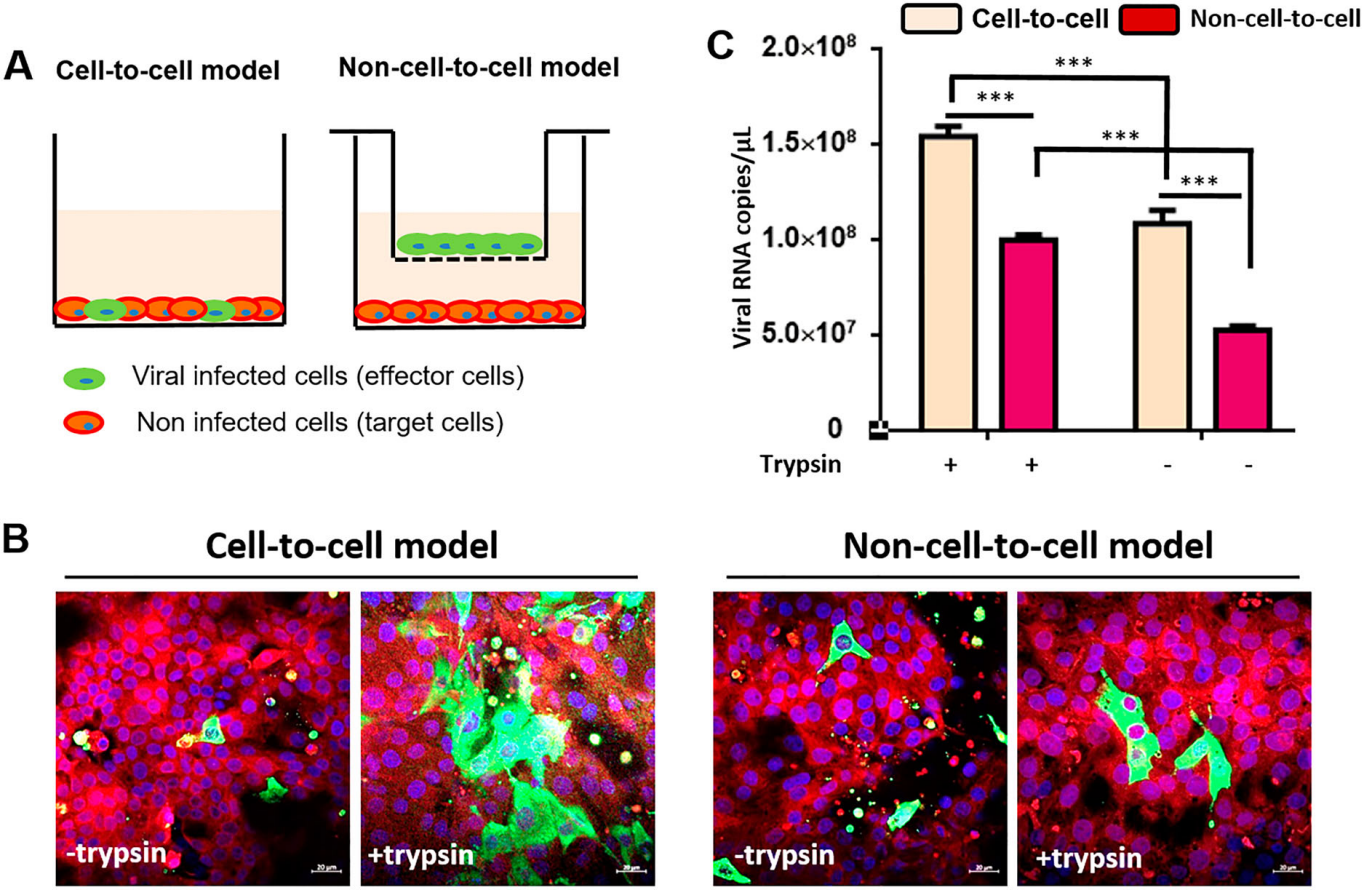
PDCoV infection spread is more efficient in a cell-to-cell manner. (A) Experimental design: PDCoV pre-infected LLC-PK cells were set as effector cells, whereas cell tracker pre-labelled non-infected LLC-PK cells were set as target cells. At 24 h post-infection, the effector cells (0.3 × 10^5^ cells) were collected and added to the target cells (1.0 × 10^5^ cells) directly (contact cell model). Or the effector cells were seeded on trans-well filters and incubated with target cells as same cell number as mentioned above (uncontact cell model). In both infection models, medium supplemented (or not) with 5 μg/ml trypsin was added. (B) After 48 h of interaction between effector cells and target cells, the expression of viral N protein in target cells were detected by immunofluorescence assay. The cell nuclei were labelled by DAPI; scale bar = 20 μm. (C) PDCoV RNA copies were quantified by qPCR in cells; error bars represent the SEM. *** stands for *p *< 0.001; experiments were repeated at least three times.

Next, we wanted to get information about the efficiency of the PDCoV spread by cell-to-cell fusion. We designed an experiment to evaluate virus replication efficacy according to two spreading models, using two distinct culture models (Figure 7(A)). The first allowed PDCoV-infected cells (effector cells) to directly interact with non-infected LLC-PK cells (target cells), referred to as the contact-cell model. The second kept the effector cells and target cells separated across the membrane of a trans-well filter, termed the non-cell-to-cell model, which allowed only free virus particles to cross the membrane and infect target cells. The results indicated that in the cell-to-cell model, with trypsin supplement, viruses transmission from effector cells to target cells was very efficient (Figure 7(B)). However, in the target cells not supplemented with trypsin, there were only a few single infected cells, and cell-to-cell fusion was rarely detected (Figure 7(B)). In the non-cell-to-cell model treated with trypsin, the cell-to-cell spread between target cells was observable, though the number and size of fusion cells was less than in the contact-cell model (Figure 7(B)). To further confirm this, PDCoV in both models with or without trypsin was quantified by qPCR, also demonstrating that PDCoV spread by cell-to-cell fusion was significantly more efficient than the non-cell-to-cell model. Furthermore, cell-to-cell spread of deltacoronavirus was slowed down if no trypsin was added (Figure 7(C)). These results indicate that PDCoV transmission via cell-to-cell spread in LLC-PK cells is very efficient.

## Discussion

Isolation and propagation of PEDV and PDCoV requires addition of exogenous trypsin to cell cultures, and thus it is commonly accepted that trypsin is essential for entry of these viruses into the cell [36–39]. However, trypsin is not indispensable for all strains of PEDV in Vero cells, as cell entry and release of the Vero cell culture-adapted DR13 (vaccine strain) was independent of trypsin [38]. For PDCoV, all previous studies have used live virus, which makes it difficult to differentiate between virus entry and the later steps of the virus lifecycle. To analyze PDCoV entry independently from other replication steps, we applied a PDCoV pseudovirus entry assay, demonstrating that trypsin failed to promote PDCoV entry in the same way as PEDV [40]. A recent study reported that PDCoV enters cells via two pathways: trypsin-mediated entry at the cell surface or cathepsin-mediated entry in the endosome [39]. Our results show that PDCoV entry does not depend on trypsin; this is consistent with the fact that PDCoV and PEDV entry is greatly activated by lysosomal proteases [39–41]. In the PEDV lifecycle, trypsin plays a crucial role in viral release [32]. However, in our study, we demonstrated that the amount of PDCoV released into the supernatant was not influenced by trypsin (Figure 2). This suggested that mechanisms of viral egress of PDCoV is different from that reported for PEDV (40).

We demonstrated that trypsin contributes to cell-to-cell membrane fusion in PDCoV infection *in vitro*, and this step needs the interaction of S glycoprotein of PDCoV and its receptor. pAPN has been reported to serve as a functional receptor for PDCoV [24,25]. However, in another study, Zhu et al. provided some evidence that pAPN may contribute to virus entry but does not serve as the primary receptor for PDCoV [42]. In this study, we found that HEK293 cells which stably express pAPN are susceptible to PDCoV infection, whereas normal HEK293 cells are resistant, supporting an important role for pAPN regardless of whether it is the primary receptor or not. Therefore, HEK293-APN cells were used in assays that could differentiate cell-to-cell fusion from other steps of the viral lifecycle. We found that trypsin mediated syncytium formation with cellular material exchange between effector and target cells.

The mechanisms contributing to the difference in cell-to-cell fusion ability of PDCoV in the different cell lines is unclear. One may speculate that variable expression of pAPN or other critical cellular factors may be responsible. Firstly, LLC-PK cells are more susceptible to PDCoV infection than ST cells under the similar condition where trypsin is supplemented in the cell culture medium, which may be one of the possible explanations for the different effects of trypsin on PDCoV replication in LLC-PK and ST cells. However, in a recent study, Zhang et al. demonstrated that the S glycoprotein could successfully induce cell-to-cell fusion in the presence of trypsin in ST cells, facilitating virus replication [39], which is contrary to our results and another previous study [28]. Hu et al. demonstrated that pancreatin rather than trypsin can promote PDCoV replication in ST cells [28], whereas our results indicated that S protein cleavage in LLC-PK cells was more pronounced than in ST cells (Figure 6(C, D)). We speculate that the ST cell line used by Zhang et al. [39] may have been a different lineage from that used in this study, possibly one with a greater receptor abundance than ours; receptor abundance is a critical switch for virus efficient replication [43]. What contributes to this difference is unclear and needs to be further studied. Trypsin promotes PDCoV replication at a late stage of the infection in LLC-PK cells, and the effect was more pronounced at low MOI (Figure 5(A, B)). This result supports our conclusion that trypsin promotes PDCoV replication at the cell-to-cell fusion stage because syncytium formation occurs at a late stage of the virus lifecycle. As determined by western blot, there was no obvious increase in viral replication in cell lysates from trypsin-treated LLC-PK cells at 12 and 24 hpi (Figure 3(A)). We think this was because a high MOI = 10 was chosen for this experiment, and the expression of viral N protein may have become saturated, making it difficult to see significant differences by western blot. In fact, when we used a lower MOI of 0.5 to inoculate LLC-PK cells, we noticed a significant increase in virus replication (Figure S1). This notion is also consistent with the finding that the promoting effect of trypsin is less pronounced at high MOI. If most cells are infected during the first round of infection, cell-to-cell spread is not required for further spread of the virus.

In summary, we identified that extracellular trypsin is required for PDCoV cell-to-cell fusion in LLC-PK cells. Based on the efficiency of infection, we also recommend isolation and propagation of PDCoV to be performed in LLC-PK cells rather than in ST cells. Furthermore, infection of LLC-PK cells should be more efficient at high confluency because it more easily allows PDCoV spread by cell-to-cell fusion. These data may provide a basis for improving virus culture methods, leading to efficient isolation and propagation of PDCoV for future development of vaccines and other therapeutic products.

## Supplementary Material

Supplemental Material
