# Supplementary material for: Trypsin promotes porcine deltacoronavirus mediating cell-to-cell fusion in a cell type-dependent manner
Source: Emerg Microbes Infect. 2020 Feb 24;9(1):457–68. doi: 10.1080/22221751.2020.1730245 (PMC7054919; doi:10.1080/22221751.2020.1730245)
Supplement: Supplemental Material [file TEMI_A_1730245_SM1138.zip › Figure legends-final.docx]

**FIGURE LEGENDS**

**FIGURE 1. Trypsin significantly promotes PDCoV replication in LLC-PK cells but not ST cells.** (**A**) LLC-PK cells were infected with PDCoV at an MOI of 0.1 in the presence or absence of 5 μg/ml trypsin, and then cells were collected at indicated time points. After cell lysis, PDCoV N proteins were analyzed by western blot. (**B**) Viral RNA was collected at 48 hpi from the experiment in (A) and quantified by qPCR. (**C**) ST cells were infected with PDCoV at an MOI of 2 in the presence or absence of 5 μg/ml trypsin, and then cells were collected at indicated time points. After cell lysis, PDCoV N proteins were analyzed by western blot. (**D**) Viral RNA was collected at 48 hpi from the experiment in (C) and quantified by qPCR. Each experiment was repeated at least 3 times. Error bars represent the standard error of the mean (SEM). *** stand for p<0.001, NS: no significant difference.

**FIGURE 2. Trypsin doesn’t affect PDCoV entry by pseudovirus or real virus.** Entry into (**A**) LLC-PK and (**B**) ST cells was tested using pseudotyped retroviruses displaying the PDCoV spike. Recombinant viruses containing luciferase were generated in HEK293T cells and then used to infect different cell lines in the presence or absence of 5 μg/ml trypsin. VSV-G pseudovirus was used as a positive control, and non-enveloped packaging group was a negative control. 24 h later, cells were washed and lysed for luciferase activity detection. Entry of real PDCoV into (**C**) LLC-PK and (**D**) ST cells was quantified by qPCR. LLC-PK and ST cells were infected with MOI=0.1 of PDCoV in the presence of the indicated trypsin concentration, and 2 h later, cells were washed and RNA was extracted and quantified by qPCR. (**E**) Cleavage status of S protein by indicated concentration of trypsin (1, 5, 10, 20 μg/ml). PDCoV virions were purified by centrifugation at 20,000 ×g for 2 h at 4 °C, and virions were incubated with the indicated concentration of trypsin at 37 °C for 2 h. N protein was used as a virus loading control. The above experiments were repeated at least 3 times. Error bars represent the SEM. NS: no significant difference.

**FIGURE 3. Trypsin doesn’t affect PDCoV release.** Release of PDCoV from **(A)** LLC-PK and **(B)** ST cells was analyzed with an MOI of 10 in the presence or absence of trypsin (5 μg/ml). The supernatant and the cell pellets were collected at 12 and 24 hpi respectively, and expression of viral N protein in both the supernatant and cell lysate was analyzed by western blot. LLC-PK cells were infected with PDCoV at MOI=0.1 **(C-D)** or MOI=1 **(E-F)** and treated with trypsin (5 μg/ml) for 24 h to increase virus replication, and the cells were further cultivated without trypsin for 48 h, then both cells were treated by indicated concentrations (5 and 20 μg/ml) of trypsin at 37 °C for 5 min. Virus was titrated in the cells **(C and E)** and supernatant **(D and F)** using plaque assay. Experiments were repeated at least 3 times. Error bars represent the standard error of the mean. NS = no significant difference.

**FIGURE 4. Trypsin promotes PDCoV-mediated cell-to-cell membrane fusion.** (**A**) LLC-PK cells were infected with PDCoV at an MOI of 1 for 2 h, washed with PBS and cultured in the presence or absence of 5 μg/ml trypsin. An immunofluorescence assay (IFA) was performed at 12 h post-infection (hpi); PDCoV N was stained in red and the cell nuclei were labeled by DAPI in blue. Scale bar = 200 μm. (**B**) HEK293-APN cells were infected with PDCoV at an MOI of 0.5 in the presence or absence of 0.01 μg/ml trypsin, and IFA was performed at 24 hpi. The PDCoV N protein was stained in red and the cell nuclei was labeled by DAPI in blue. Scale bar = 400 μm. (**C**) PDCoV spike-mediated cell-to-cell membrane fusion was studied in the presence of trypsin. HEK293-APN cells were co-transfected with pBind-Id and PACT-Myod and mixed with other HEK293-APN cells co-transfected with PDCoV spike and PGL5-Luc. After attachment, cells were co-cultured in fresh media containing 10 or 50 ng/ml trypsin, or no trypsin (NC). After 48 h, cell-to-cell membrane fusion was evaluated using luciferase activity; *: p<0.05 (t-test). Experiments were repeated at least 3 times.

**FIGURE 5. LLC-PK cells were more susceptible to PDCoV infection than ST cells.** LLC-PK cells were infected with PDCoV at an MOI of (**A**) 0.5, (**B**) 1, or (**C**) 10, and ST cells were similarly infected at an MOI of (**D**) 1, (**E**) 2, or (**F**) 5. Both infected cell types were cultured in the presence or absence of 5 μg/ml trypsin and then cells were washed and lysed for western blot at 8, 12 and 24 hpi. PDCoV N proteins were analyzed with a specific antibody against N protein, and actin was used as a loading control. Experiments were repeated at least 3 times.

**FIGURE 6. Spread of PDCoV was different in LLC-PK and ST cells.** (**A**) LLC-PK and (**B**) ST cells were infected with PDCoV at a low MOI (MOI=0.01) with trypsin (5 μg/ml), then samples were fixed and IFA performed at 48 hpi. The PDCoV N protein was stained in red and the cell nuclei was labeled by DAPI in blue. Arrows indicate syncytium formation; scale bar = 200 μm. Cleavage status of S protein in (**C**) LLC-PK cells and (**D**) ST cells by indicated concentration (5, 50, 100, 200 μg/ml) of trypsin. LLC-PK cells were infected in the presence of trypsin with PDCoV (MOI=0.1), whereas ST cells were infected with PDCoV (MOI=10) for 24 h. In order to increase virus replication and bring S protein to a detectable level, the cells were further cultivated without trypsin for 24 h, then both cells were treated by the indicated concentration (5, 50, 100, 200 μg/ml) of trypsin at 37 °C for 2 h. N protein and actin were used as a virus loading control. Experiments were repeated at least 3 times.

**FIGURE 7. PDCoV infection spread is more efficient in a cell-to-cell manner.** (**A**) Experimental design: PDCoV pre-infected LLC-PK cells were set as effector cells, whereas cell tracker pre-labeled non-infected LLC-PK cells were set as target cells. At 24 h post-infection, the effector cells (0.3×10^5^ cells) were collected and added to the target cells (1.0×10^5^ cells) directly (contact cell model). Or the effector cells were seeded on trans-well filters and incubated with target cells as same cell number as mentioned above (uncontact cell model). In both infection models, medium supplemented (or not) with 5 μg/ml trypsin was added. (**B**) After 48 hours of interaction between effector cells and target cells, the expression of viral N protein (green) in target cells (red) were detected by immunofluorescence assay. The cell nuclei were labeled by DAPI in blue; scale bar = 20 μm. (**C**) PDCoV RNA copies were quantified by qPCR in cells; error bars represent the SEM. *** stands for p<0.001; experiments were repeated at least 3 times.
