# Supplementary material for: Trypsin promotes porcine deltacoronavirus mediating cell-to-cell fusion in a cell type-dependent manner
Source: Emerg Microbes Infect. 2020 Feb 24;9(1):457–68. doi: 10.1080/22221751.2020.1730245 (PMC7054919; doi:10.1080/22221751.2020.1730245)
Supplement: Supplemental Material [file TEMI_A_1730245_SM1138.zip › FIGURE S legend.docx]

**FIGURE S1. Trypsin doesn’t affect PDCoV pseudovirus release.** Release of PDCoV from **(A)** LLC-PK were analyzed with an MOI of 0.5 and **(B)** ST cells were analyzed with an MOI of 5 in the presence or absence of trypsin (5 μg/ml). The supernatant and the cell pellets were collected at 12 and 24 hpi, and expression of viral N protein in both the supernatant and cell lysate was analyzed by western blot.
